# The duality of hope and challenges: a phenomenological study of first-year university students’ experiences in South Africa

**DOI:** 10.3389/fpsyg.2024.1470943

**Published:** 2024-11-21

**Authors:** Henry D. Mason

**Affiliations:** Tshwane University of Technology, Pretoria, South Africa

**Keywords:** black tax, counselling units, hope, interpretative phenomenological analysis, south African students, student affairs

## Abstract

**Introduction:**

In South Africa, access to higher education is viewed as a pathway to improved life chances. However, the transition from school to university is a stressful experience for students, marked by significant challenges. Although literature acknowledges these challenges, limited research has focused specifically on the role of hope during this period. Additionally, previous studies on hope have often utilized individualistic approaches, which may not fully capture the experience of students in collectivist cultures. This study addresses this gap by exploring hope from a culturally sensitive perspective within a collectivist context, aiming to understand how first-year South African university students experience hope during the transition to university.

**Methods:**

This study employed Interpretative Phenomenological Analysis (IPA) to explore the lived experiences of hope among first-year South African university students during their transition to higher education. Twenty-two students participated in semi-structured interviews, allowing for an in-depth examination of their personal and cultural perspectives on hope and the challenges they faced. The data were analyzed iteratively, with strategies implemented to enhance trustworthiness and credibility, ensuring a thorough interpretation of students’ experiences.

**Results:**

The analysis yielded three main themes: (1) Affective and Social Duality: Students described mixed emotions and social challenges as they entered university, highlighting the dual nature of their experiences in adapting to a new environment. (2) Hope as a Multifaceted Concept: Hope was portrayed as a guiding force that helped students navigate periods of uncertainty. Participants described hope not just as a single idea but as a complex, evolving concept crucial to their resilience. (3) Beyond Academic Aspirations: Hope extended beyond academic success and was closely tied to personal fulfilment and the desire to contribute positively to society. This broader perspective on hope suggests that students’ aspirations were not confined to individual achievement but also included a sense of collective and societal impact.

**Discussion:**

The study reveals that hope is a multidimensional construct that significantly influences students’ transitional experiences, extending beyond academic goals to include personal growth and societal contributions. This finding challenges the traditional, individualistic approaches to studying hope by highlighting the cultural relevance of collectivist values in shaping students’ experiences. The study underscores the need for culturally sensitive research and suggests that student support services should consider cultural contexts to address students’ unique challenges and aspirations better. Future research is recommended to explore hope across various cultural backgrounds to gain a more comprehensive understanding of its role in student development.

## 1 Introduction

Dreaming of and realising a brighter future has moved humanity forward for centuries. An illustration of such a vision includes Nelson Mandela’s journey, documented in his autobiography *Long Walk to Freedom*, which foresaw a rainbow nation celebrating racial equality and freedom (Mandela, 1995). At the heart of Mandela’s vision, we find the principles central to hope theory, namely that the future can be better than the present, and people can make it so (Lopez et al., 2003; Snyder et al., 2002). The principles of hope theory also resonate with university students, who view higher education as a crucial enabler of life opportunities and a pathway to a brighter future (Cherrington, 2018; Guse and Vermaak, 2011; Mason, 2020). Hope inherently carries a duality; it thrives on envisioning a brighter future but often exists in tension with the constraints imposed by diverse challenges. In the context of South African university students, this tension becomes especially pronounced during their complex transition to university life. A comprehensive understanding of hope necessitates examining the lived experience amid students’ adversities, including the transition to university. This study seeks to explore hope specifically in relation to the psychological distress and challenges faced by first-year university students in South Africa.

Seeking a better life is particularly relevant in South Africa. Amongst other things, South Africa has an unemployment rate of 32.90%, disproportionately higher at 45.50% among youth (persons aged between 15 and 34 years). However, national statistics also indicate that higher-education graduates experience lower unemployment, as their skills offer a gateway to the world of work (Statistics South Africa, 2022). Hence, while youth bear the brunt of unemployment in South Africa, those who gain access to and complete a university qualification experience greater social and economic mobility, which could be considered the cornerstone of building an economically emancipated life (Du Plooy and Zilindile, 2014). Although obtaining a higher education qualification significantly enhances employability and economic mobility for South African youths, this process begins with a critical transition.

The transition from school to university is a critical developmental period in young people’s lives (Cameron and Rideout, 2020). This transition is characterised by, *inter alia*, developmental, emotional and physical changes and academic stressors (Arnett, 2015; Fennie et al., 2020; Maniram, 2022). Not only are first-year university students busy forming identities, cultivating friendships, dealing with a changing environment, and managing high academic workloads, but they also struggle with epistemic access (Lewin and Mawoyo, 2014; Tinto, 2009; Xulu-Gama and Hadebe, 2022).

Epistemic access, which refers to students’ ability to engage with, understand, and use knowledge within the educational context, is particularly troubling in South Africa because students’ linguistically diverse backgrounds can create barriers to accessing and benefiting from higher education (Lewin and Mawoyo, 2014). Socioeconomic factors such as financial stressors, unemployment, and being first-generation students compound the challenges associated with epistemic access (Moosa and Langsford, 2021; Van Wyk et al., 2022; Van Zyl, 2016).

A first-generation student is the first in their family to attend an institution of higher learning, such as a university (Toutkoushian et al., 2021). Research indicates that first-generation students encounter significant stressors as they enter higher education, including financial pressure, a lack of familial support or understanding of the university, and accompanying academic challenges (Amirkhan et al., 2023; Lewin and Mawoyo, 2014; Scott, 2018; Toutkoushian et al., 2021).

The combination of developmental tasks, epistemic access, socioeconomic challenges, and first-generation students can negatively affect students’ well-being, exacerbate psychological distress, and affect their academic performance (Bantjes et al., 2023; Fennie et al., 2020; Maniram, 2022; Van Zyl, 2016). In light of these complex challenges, it is critical that university staff, such as student affairs professionals, empathise with students’ lived experiences as they affect and shape their engagement in the university context (Rimm-Kaufman and Hamre, 2010). Moreover, student affairs professionals should actively seek ways to support students, thereby enhancing student success (Blokland and Kirkcaldy, 2022; Eloff and Graham, 2020; Lai et al., 2024). Student success is a holistic concept that encompasses academic performance and psychological well-being and equips students with the skills, knowledge, and attitudes necessary to engage in the world of work and beyond as responsible citizens (Coetzee and Schreuder, 2021; Olugbara et al., 2023; Sinclair, 2019). Understanding these dynamics is crucial to framing hope as a response to challenges and a guiding force that helps students navigate their transformative educational journey amid adversity.

Research has highlighted the importance of cultivating hope among students to enhance success in the face of academic challenges (Marques et al., 2017; Rand et al., 2011). However, little is known about how first-year students, particularly those in non-Western cultures, deal with the conflicting forces of hope and adversity (Amirkhan et al., 2023; Cherrington, 2018; Marsay, 2020; Mason, 2020). This qualitative study explored the complex interplay between the hopes and challenges of first-year university students in South Africa, shedding light on how they navigated the transition from school to university. Furthermore, this study sought to develop an understanding of students’ conceptualisation of hope. Finally, the study focused on identifying principles that could be utilised in student affairs to support students in cultivating hope.

### 1.1 The duality of hope and challenge amidst the first-year experience

Existing literature has documented first-year students’ challenges, including psychological distress, academic stressors, and socioeconomic vulnerabilities (Cameron and Rideout, 2020; Lai et al., 2024; Maniram, 2022; Toutkoushian et al., 2021; Van Zyl, 2016). In light of these challenges, research has also highlighted the importance of cultivating hope as a critical factor in supporting student success directly and indirectly (Guse and Vermaak, 2011; Mason, 2020; Snyder et al., 2002).

Theorists agree that hope is more than platitude and hedonic positivity (Lopez, 2013; Mason, 2020). Instead, hope encapsulates a cognitive process positively associated with a series of affirming life outcomes, ranging from well-being to resilience to academic success (Onwuegbuzie and Snyder, 2000; Polat and Aliyev, 2024). Furthermore, they propose a two-factor model of hope, which could be trait and state-like, comprising agency (WillPower) and pathways thinking (WayPower) (Colla et al., 2022; Lopez et al., 2003; Snyder, 2002).

WillPower and WayPower are positively associated yet distinct in that a person may report high levels of WillPower or agency and low levels of WayPower or pathways thinking. However, the cumulative effect of agency and pathways thinking creates a dynamic motivational force that can propel people forward towards pursuing and achieving grand life visions (Snyder, 2002). Accordingly, a third component, namely, a vision, is relevant to describing the concept of hope.

The vision component refers to an individual’s ability to envision a positive future and imagine their best selves attaining their goals (Bulley, 2018; King, 2001). Envisioning a meaningful future aids in establishing a dynamic motivational tension between a person’s current and ideal future state (Frankl, 2006; Shantall, 2003). This dynamic tension is a metaphorical anchor that can pull a person towards an envisioned future by incorporating agency and the ability to identify and engage in various pathways (Frankl, 2006; Shantall, 2003).

The pathways component involves people’s capabilities to remain agile in the face of challenges and devise routes to their desired goals (Lopez, 2010). Hence, pathways thinking is intimately linked to the capacity to solve problems, which are part and parcel of students’ university experiences. Students’ capacity to deal with difficulties proficiently is linked to enhanced academic outcomes and better emotional regulation (Bieleke et al., 2020). Student agency is essential to drive pathways thinking (Snyder, 2002).

Agency refers to the motivation and drive to initiate and continue efforts towards goal attainment (Marques et al., 2017). Proponents of goal-setting theory argue that the capacity to initiate and take sustained action towards a set of goals is a critical determinant of goal achievement (Latham and Locke, 2007). They also emphasise that goal setting, or envisioning a desired future state must be accompanied by consistent motivation and concrete action if the aim is to move from merely fantasising about the future and achieving specific outcomes (Bieleke et al., 2020; Locke and Latham, 2002).

Snyder (2002) and Lopez (2013) hope model has been criticised due to its individualistic focus because it fails to encompass values espoused in collectivist cultures (Bernardo, 2010; Cherrington, 2017; Colla et al., 2022; Du and King, 2013). Furthermore, research on hope, especially among student populations, has predominantly been articulated from a positivistic perspective drawing on psychometric instruments developed in Western contexts (Krafft et al., 2023). Such instruments perpetuate Western conceptualisations, encouraging an individualistic focus (Cherrington, 2017; Colla et al., 2022). Additionally, current models of hope theory commonly focus on individual agency and achievement, overlooking how communal values and socio-cultural factors shape the psychological experiences of hope in collectivist cultures. This gap raises concerns about the applicability of these theories to understand how hope influences university students in South Africa’s collectivist context (Colla et al., 2022; Mason, 2020). Hence, there is a need to explore hope from other methodological perspectives, such as qualitative inquiries, to develop a deeper understanding of the intricacies of hope as experienced by students from, *inter alia*, non-Western and collectivist perspectives (Bernardo, 2010; Eren and Yeşilbursa, 2017; McNulty and Fincham, 2012).

A collectivist culture refers to a societal framework where people are closely integrated into cohesive groups, for example, extended families and communities, which focus on the group versus the individual and provide collective social protection and support (Heu et al., 2019). In collectivist groups, the dominant desires and expectations of the group take precedence over those of the individual (Shiraev and Levy, 2020; Sue et al., 2023). Collectivist values, such as social harmony and shared identity, contrast values espoused in individualistic cultures, such as self-actualisation and independence (Heu et al., 2019; Shiraev and Levy, 2020).

The integrated theory of hope proposed by Scioli et al. (2011) suggests that hope is a universal human experience shaped by diverse cultural factors, including collectivism. The African philosophy of Ubuntu underscores the interconnectedness among people, suggesting that hope must be seen not merely as an individual desire but as a communal force that fosters resilience and support within social networks, thus offering a more nuanced understanding of hope in collectivist contexts (Broodryk, 2006; Letseka, 2012). In collectivist cultures, the experience and expression of hope may differ from their manifestation in individualist contexts (Broodryk, 2006; Sue et al., 2023). Colla et al. (2022) add that hope ought to be expanded to include an intrapersonal motivational component (WhyPower) and an interrelational and socio-contextual aspect (WePower). Thus, hope emerges from an ecologically nested system, and various perspectives should be considered (Colla et al., 2022).

In light of the above, Cherrington (2017) proposed an African conceptualisation of hope adapted from Scioli’s integrated hope theory (Scioli, 2007). Scioli’s theory views hope as a multifaceted construct, incorporating cognitive, emotional, spiritual, and motivational dimensions (Scioli, 2007; Scioli and Biller, 2010; Scioli et al., 2011). It combines perspectives from psychology, philosophy, and biology to understand hope comprehensively. Building on this foundation, Cherrington (2017) introduces an Afrocentric perspective, framing hope as a future-oriented, multilayered, and multidimensional experience, structured around three subsystems: attachment, mastery, and survival.

A notable feature of Cherrington’s model is its hierarchical framework of hope development, progressing across six levels: (1) biological motives (innate human drives), (2) contextual hope (hope within various contexts), (3) personal hope (individual beliefs), (4) belief systems (cultural values and norms), (5) relational hope (hope as it relates to others), and (6) collective hope (emphasising the group over the individual). While this model offers a culturally enriched view of hope, particularly through its integration of collective values, it raises the issue of interconnectedness that many mainstream hope theories, often rooted primarily in Western individualistic paradigms, often fail to capture sufficiently.

In collectivist cultures, hope is deeply intertwined with communal values, social interconnectedness, and collective well-being rather than solely centred on individual aspirations (Scioli, 2007). The focus on relational hope and collective hope within Cherrington’s framework acknowledges that in collectivist societies, the experience of hope is not isolated to the personal domain; it is significantly shaped by one’s relationships and the group’s overall success (Cherrington, 2017) This aspect challenges the more individualistic focus of many Western theories of hope, which often prioritise personal mastery and self-actualisation over collective goals (Heu et al., 2019; Shiraev and Levy, 2020; Snyder et al., 1997).

Cherrington’s emphasis underscores the unique ways in which hope operates in collectivist societies, where the group’s well-being is paramount, and individuals derive hope from the success and harmony of their community. From this perspective, hope becomes a shared, communal experience, which may explain why current theories steeped in an individualistic perspective fail to adequately address the hope experienced in collectivist settings (Colla et al., 2022). By highlighting these distinctions, Cherrington’s Afrocentric model offers a more culturally nuanced perspective, addressing the gap in hope theories that often overlook the collective dimensions of human experience. This calls for a critical examination of how hope frameworks can be expanded to better account for the communal, relational aspects of hope prevalent in non-Western, collectivist cultures (Cherrington, 2017). Another crucial dimension often overlooked in mainstream theories is the role of religiosity and spirituality (Mason, 2020). These elements are intrinsically connected with the collective experience of hope, particularly in African contexts, where spiritual beliefs significantly influence individuals’ perceptions of hope and well-being (Broodryk, 2006; Nell, 2016; Van der Merwe et al., 2010).

Religiosity and spirituality play essential roles within African and, specifically, South African cultures. Spiritual beliefs and religion serve as pillars of support and guidance for many individuals, including university students in South Africa (Nell, 2016). Consequently, religiosity and spirituality can influence students’ experiences and conceptions of hope because they serve as essential paradigms in making sense of the world and coping with challenges during stressful and uncertain times (Van der Merwe et al., 2010). The religious and spiritual dimensions add to the multidimensional conception of hope within non-Western cultures (Colla et al., 2022; Scioli et al., 2011).

Another crucial aspect that merits examination is the role of student affairs in supporting first-year students in cultivating hope and addressing academic stressors (Cherrington and De Lange, 2016; Eloff and Graham, 2020; Olugbara et al., 2023). Although research has focused on the challenges confronting first-year students, there is a paucity of literature, especially within African and South African contexts, on how universities can leverage hope theory to provide tailored support and interventions (Cherrington, 2017, 2018; Colla et al., 2022; Guse and Vermaak, 2011; Mason, 2020). Exploration among first-year students can provide insight on how student affairs professionals can leverage hope and offer relevant support initiatives to empower and guide first-year students through their academic journeys.

In conclusion, this review of the literature indicates that the concept of hope is rich with theoretical depth, encompassing both Western and African perspectives. Western theories, particularly Snyder’s hope theory, define hope from a cognitive perspective, emphasising individual agency, and as a mechanism that can enhance resilience and academic success (Lopez et al., 2003). However, in African societies, hope is deeply interconnected with cultural values like Ubuntu, emphasising social harmony, interdependence, and shared identity (Broodryk, 2006; Letseka, 2012). The Afrocentric perspective regards hope not only in relation to personal success but also in terms of collective upliftment and the responsibility to family and community. This study, therefore, enriches the understanding of hope by bridging these perspectives, recognising hope as a universal experience with culturally specific manifestations that hold potential for broad societal impact.

### 1.2 The present study

This qualitative study, adopting a phenomenological design, explored first-year South African university students’ conceptions of hope as they transitioned from school to the university context. The study was guided by the following overarching research question: What are first-year South African university students’ qualitative conceptions of hope and challenge within the university context?

## 2 Method

### 2.1 Research approach and strategy

Interpretative Phenomenological Analysis (IPA) was adapted to explore students’ qualitative conceptions of hope (Brocki and Wearden, 2006; Smith et al., 2009). The primary aim of IPA is to explore how persons make sense of specific phenomena. IPA acknowledges that humans are sense-making beings who interpret the world from their unique vantage points. Hence, IPA allows for a deep, empathetic, nuanced exploration and sense-making activity of people’s lived experiences (Brocki and Wearden, 2006; Smith et al., 2009).

A phenomenological approach, such as IPA, is particularly suited for exploring how hope and adversity are experienced by specific subgroups, such as first-year university students in South Africa, because it can provide rich and in-depth insights into their lived experiences. The transitional period from school to university is marked by developmental challenges and significant socioeconomic and cultural pressures, including those tied to the collectivist values prevalent in South African society.

Existing frameworks may not fully capture the nuanced ways in which hope is both a personal and collective experience for these students (Colla et al., 2022). The phenomenological method also explores how hope is experienced at this intersection of academic, familial, and societal pressures. It could, therefore, assist in illuminating how hope functions as a dynamic and multifaceted resource, guiding students through individual and collective adversities.

By focusing on this specific subgroup, a phenomenological approach can offer insight into how hope operates within the context of first-year students, making visible the resilience and challenges deeply embedded in their unique socio-cultural realities. This inquiry is thus essential to extending our understanding of hope beyond abstract theories, grounding it in the lived experiences of a vulnerable and transitional population.

### 2.2 Participants and setting

The study was conducted at a residential, public South African metropolitan university that enrols approximately 40,000 students annually. The university offers a variety of academic programmes, and the student population reflects the demographics of the South African population.

Invitations to participate in the research were sent to first-year students who attended a psychoeducational support programme. Twenty-two first-year students volunteered to participate in this study. The students’ ages ranged from 18 to 23, and 12 identified as female and 10 as male. The sample size was deemed appropriate because the study aimed to develop a general understanding of students’ experiences regarding hope and challenge versus an in-depth analysis of any particular case. Furthermore, the participants represented a group for whom hope and challenge were relevant as they navigated the transition from school to university (Creswell and Poth, 2018).

### 2.3 Data collection and procedure

Two fieldworkers, both postgraduate students, collected data for this study. The fieldworkers were briefed regarding the aims of the study and received training to collect the data. The training included information on research ethics, IPA, qualitative data collection using interviewing, and hope theory. The fieldworkers signed agreement forms indicating that the content of the interviews would be kept private and that concerns regarding their well-being would be reported to the researcher. Following the interviews, the fieldworkers were debriefed.

Of primary concern when conducting IPA studies is the requirement to collect rich data from participants (Creswell and Poth, 2018). Accordingly, semi-structured individual interviews were used as they enabled the fieldworkers and participants to have a phenomenologically-based conversation in real time while allowing the opportunity to explore new material that may arise (Creswell and Poth, 2018). Consequently, the interviews were designed to capture a comprehensive understanding of the participants’ experiences, the significance of hope in their journeys, the challenges experienced, and the support systems that facilitate their success.

The interviews focused on allowing students to tell their stories about hope and challenge rather than checking preconceived notions. The following four questions framed and guided the interview process: (1) Can you tell me about your experience of transitioning from school to university? (2) What does hope mean to you? (3) What role has hope played, if any, during your university journey thus far? (4) Would it be relevant for the university to assist students in cultivating hope? Additional probes (e.g., Can you tell me more?) and prompts (e.g., Can you provide an example from your life to illustrate the experience you shared?) were used to delve more deeply into pertinent issues. The interview duration was 29 to 61 min, averaging 41 min per interview. All interviews were audio-recorded and transcribed verbatim. The average interview transcription was 12 pages in length and, in total, comprised 255 pages.

### 2.4 Data analysis and trustworthiness

This section details the processes used in qualitative data analysis for this study and the measures taken to ensure trustworthiness. The researcher conducted the qualitative analysis. To enhance trustworthiness, an external coder provided an additional layer of validation and confirmed the accuracy and reliability of the findings.

The transcribed interviews served as the primary data and were analysed using IPA (Brocki and Wearden, 2006; Smith and Osborn, 2008). As qualitative researcher, I employed bracketing through qualitative memo writing to document personal reflections and experiences regarding the phenomenon of hope in the transition to university. This approach facilitated systematic self-reflection, wherein I noted professional and personal insights, such as career-related uncertainties, decision-making conflicts regarding my entry higher education studies, how I utilised social support networks to manage transition-related stress, and my academic background and studies regarding the first-year experience. I recorded reactions, thoughts, and potential preconceptions throughout data immersion, allowing for disciplined subjective engagement with participants’ experiences. This bracketing process helped maintain a balanced perspective between emic interpretations—understanding hope through theoretical frameworks—and etic observations, examining the process with an outsider’s view. The analysis followed five iterative and interconnected steps to ensure a rigorous, reflective process (Creswell and Poth, 2018).

First, I immersed myself in the data by debriefing the fieldworkers and considering their perspectives on the interview process. I also listened to the audio recordings and re-read the transcripts multiple times. I then engaged in qualitative memoing to reflect on thoughts and ideas that emerged from the data. Second, the data were coded by assigning descriptive labels to portions of the text, thereby capturing and summarising the essence of the participants’ responses. The initial coding process aimed to create a detailed albeit distilled set of codes reflecting higher-order conceptualisation. Third, the codes were systematically compared, contrasted, and organised into themes and subthemes. This step involved grouping similar codes to form common patterns across the datasets. The process of theme development allowed higher levels of conceptualisation and abstraction, thereby moving beyond mere description to identifying underlying patterns in participants’ phenomenological experiences.

Fourth, the emergent themes were re-examined, grouped, and labelled. This iterative process involved constant refinement and re-evaluation to maintain coherence and relevance. The final step entailed writing up and presenting the qualitative interpretation, thereby situating the findings within the context of the existing literature while retaining the flexibility to include novel and new insights.

After analysing 17 interviews, data saturation was reached, indicating that no new significant information emerged. However, to ensure robustness and confirm the stability of the findings, all 22 interviews were analysed, ensuring that no new themes emerged and that the data thoroughly supported the existing themes.

Various strategies were implemented to ensure the trustworthiness and credibility of the qualitative analysis (Creswell and Poth, 2018; Lincoln and Guba, 1985). First, an external qualitative coder studied the initial three transcripts and codes. Subsequently, the two readings of the accounts were discussed to reach an agreement on a warrantable, and not necessarily definitive and singular, account of the data. The external coder also considered the final account and confirmed that the qualitative representation represented a fair and accurate interpretation of the data. Second, the analysis and subsequent interpretation were cross-checked against a literature control. Third, participants verified the interpretation as an accurate account of their perspectives. Finally, qualitative memoing was conducted to reflect on the analytic procedures (e.g., the qualitative analysis), theoretical considerations (e.g., linking themes to theoretical perspectives), and personal experiences (e.g., thoughts about the qualitative process).

## 3 Findings and discussion

Three superordinate themes emerged during the qualitative analysis: (1) Affective and social duality in first-year university experiences, (2) hope as a multifaceted concept, and (3) future aspirations and culture. The referencing system used to qualify the verbatim quotes in parenthesis denotes participant numbers (e.g., P#1 for Participant 1), age (e.g., 20 indicating 20 years of age), and sex (M = male, F = female). To enhance the readability of the participant quotes, basic grammatical and syntax errors were corrected without changing the meaning of participants’ responses.

### 3.1 Affective and social duality in first-year university experiences

This theme captures the intricate, affective, and social dualities first-year students face as they enter university. The students’ narratives revealed a contrast between pride and joy alongside apprehension and struggle, shaping their journeys through positive and challenging experiences. Specifically, students expressed pride in their achievements and the joy felt by their families: “*My family was so encouraged to have a daughter, the first female in the family… admitted to the university. It was a celebration*” (P#10, F, 20). However, this optimism was often accompanied by significant financial concerns, highlighting a common financial burden of higher education. For instance, one participant explained, “*I could only afford it through NSFAS [bursary scheme in South Africa]… there wasn’t another way*” (P#14, M, 18).

Alongside financial concerns, students felt an overarching sense of uncertainty and self-doubt: “*It is stressful… feeling I’m not good enough, what if I fail?*” (P#12, F, 23). This complex combination of emotions underscores the pressures of being a first-generation student, including the lack of familial guidance, as described by one student: “*Being the first one [first-generation student] was good but also difficult. No one understands… no one gave advice or helped me cope*” (P#8, F, 19).

The data further indicated a broader spectrum of emotional experiences, ranging from excitement (“*It is so great to be a university student*” P#20, M, 19) to anxiety when confronting the unfamiliar environment (“*Everything was so new… I found the people and the buildings strange… very overwhelming*” P#5, F, 18). Students also expressed a sense of responsibility toward others, viewing their education as a potential example for their communities: “*If I can be an example to others, they will find it easier… others like young people who struggled in life*” (P#3, F, 19). This blend of hope and challenge highlighted an affective duality, where students navigated social expectations within their family and broader community.

In line with previous studies, this qualitative theme confirms that university life imposes a unique blend of academic, social, and societal expectations on first-year students (Fennie et al., 2020; Lewin and Mawoyo, 2014; Lai et al., 2024). The need to adapt to a new environment, developmental challenges, and social integration underscores the overwhelming nature of this transition (Arnett, 2015; Van Wyk et al., 2022). In this regard, Participant 13 shared how daunting the adjustment felt: “*… it was hard… there were so many new things to learn, and the people were not helpful… felt lost and experienced lots of stress*” (P#13, M, 18).

Despite these challenges, many students noted a positive transformation over time, where initial stress evolved into resilience and empowerment. Participant 7 reflected, “*It was stressful at the start, but I grew [especially] when I think back to the start of university and where I am now*” (P#7, F, 19). Another participant highlighted newfound strength and academic growth: “*I am stronger and more academically fit than I was in January when I arrived here*” (P#3, F, 22). Over time, students reported feeling more directed, with goals becoming clearer (“*I am more sure now of where my life will go*” P#18, M, 20). Thus, the initial phase of adjustment, though intense, laid the foundation for empowerment as students engaged with resources, social networks, and self-developed strategies to handle academic and personal responsibilities (Mason, 2020).

In summary, this affective and social duality—the oscillation between hope and pressure—requires resilience from students as they transition from initial overwhelm to a sense of purpose and belonging. This theme encapsulates the first-year journey of emotional oscillation, with students transforming early challenges into meaningful experiences that contribute to their growth and adaptation in higher education.

### 3.2 Hope: a multifaceted concept

The students described hope as a multifaceted concept encompassing four interrelated components, categorised as follows: (1) hope as a guiding light in times of uncertainty, (2) fostering a sense of belonging and confidence, (3) a source of strength when confronting obstacles, and (4) hope and religion. The various facets are briefly discussed as subthemes below.

#### 3.2.1 Hope as a guiding light

Participants described hope as a “*light in the darkness*” (P#4, F, 20), providing direction and clarity when facing academic setbacks or feeling overwhelmed. Additionally, students indicated that hope fostered resilience (“*I tell myself to keep believing and I will get the strengths to make it*” P#16, M, 19), allowing them to persevere when encountering academic and other challenges (“*I say to myself ‘Just keep going and believe you can do it’. I realised I must be my own supporter and believe in myself*” P#14, M, 18). When viewed through the lens of hope theory, this subtheme points to the relevance of a guiding vision that can serve as consolation and encouragement when students encounter the stressors that are part and parcel of the university experience (Lai et al., 2024; Lopez, 2013).

#### 3.2.2 Hope to foster a sense of belonging and confidence

The influence of hope extended beyond individual pursuits and interests and entailed engagement with others in various contexts. Participants who engaged in relational activities, for example, as members of sports and cultural groups, reported a stronger sense of belonging, which, in turn, reinforced their hope and self-confidence. In this regard, participant 14, an 18-year-old male, reflected, “*Being part of the cricket club and playing sports provides camaraderie. You feel supported and cared for. Others ask about your academics and social life. It makes me feel part of the university*.” Cherrington (2017) highlighted the importance of relational hope that develops in relationships with others. Likewise, Colla et al. (2022) draw attention to the notion of WePower. In this regard, numerous participants pointed to relational hope and emphasised its positive effects on their academic-related behaviour (“*We have a tutor group and support each other very much. It helps with studying and dealing with stressors like missing class or completing a lot of online work*” P#4, F, 20). This finding highlights the importance of relational and communal engagement in nurturing hope and subsequent positive outcomes. Engaging in activities that foster a sense WePower could strengthen WillPower and WayPower (Cherrington, 2017; Colla et al., 2022; Mason, 2020).

#### 3.2.3 Hope as a resilient force and motivational catalyst in navigating university challenges

In the challenging landscape of university life, hope emerges as a multifaceted source of strength and motivation, empowering students to confront diverse obstacles while sustaining a forward-looking vision. Faced with issues ranging from gender-based violence and academic overload to social isolation, students demonstrate resilience by drawing from hope in various forms. As one student noted, the pervasiveness of gender biases, both within and beyond university settings, requires resilience and honesty: “*GBV [gender-based violence] is big in the country, not just at universities … it will become safer for females out there, but we need to be strong and deal with it honestly*” (P#12, F, 23). Others reflected on struggles with inclusion and self-doubt, as voiced by a young male participant: “*For me, it is hard when I feel left out… not included and it makes me doubt myself*” (P#15, M, 19). These experiences reveal a common thread where hope becomes an essential coping resource that empowers students to navigate difficult moments with a renewed sense of agency and purpose.

Students described various sources of hope that strengthen their resilience in the face of adversity, from personal work ethics (“*My work ethic is strong and gives me hope*” P#13, M, 18) to creative outlets like music and poetry (“*I love music and writing poetry… helps me cope better*” P#9, F, 19). This expression of hope aligns with Frankl’s (2006) concept of meaning, attained through actualising values tied to creative expression, experiential engagement, and personal attitude. Shantall (2003) further emphasises that realising meaning-centred values amid challenges promotes a sense of purpose and hope, driving students to overcome obstacles while envisioning a better future.

In addition to resilience, hope serves as a motivational catalyst that fuels students’ ability to adapt to the demands of higher education. For example, one student noted, “*Believing that I can be somebody, achieve great things, even not-so-great things, but be successful… is what helped me deal with stressors*” (P21, M, 18), highlighting how hope propels them toward self-actualisation. This forward-looking stance also aids in forming supportive social networks: “*The ministry I belong to helped with making friends, and they were a great support when I came here*” (P#17, M, 18). Thus, hope not only aids in building resilience but also enhances social bonds that fortify students’ support systems.

As students articulate their goals and envision a promising future, hope is a pivotal resource for managing self-doubt and embracing personal growth. One participant, for instance, reflected on overcoming insecurities through a process of self-affirmation and goal-setting: “*I read a book about a doctor who said you must upgrade your self-esteem, how you see yourself, and follow your plan to achieve bigger results*” (P#7, F, 19). Similarly, another student explained, “*Becoming a better version of who I am… that is what I hope for and work for*” (P#13, M, 18). Such reflections reveal hope as more than an emotion; it is an intentional stance and belief in the possibility of success even in challenging circumstances: “*Hope is the belief that I can do something even when it is hard*” (P#12, F, 23). This perspective echoes Frankl’s (2006) idea that meaning is derived from one’s attitude in confronting life’s stressors, making hope a foundational element of students’ resilience and growth.

#### 3.2.4 Hope and religion

Religiosity also emerged as an essential source of hope. It was described by participants as central to their lives (“*My relationship with Christ is very important, and it is my sense of power*” P#6, F, 18) and a source of solace during difficult times (“*When things get very difficult I pray and turn to God*” P#11, M, 19). The combination of religion and social support through church meetings and cell groups also emerged as a critical element in promoting hope among the students (“*We have cell group on Thursdays, and it means a lot to talk about my beliefs, pray and sing with others about something so close to my heart*” P#9, F, 19). This finding relates to previous South African research highlighting the importance of religion and spirituality in the lives of South African university students and others (Nell, 2016).

### 3.3 Future aspirations and culture

This theme highlights the intersection between students’ future aspirations, the role universities, specifically counselling centres, can play in shaping those aspirations, and the influence of culture. The qualitative data revealed that academic achievements are a significant part of students’ future ambitions. For example, participant 20 indicated, “*My goal is to graduate with honours and that my degree helps to achieve success and financial independence*” (M, 20). Other participants agreed that attaining academic qualifications would serve as avenues to more empowered lives “… *having this qualification will help me be a person who can create a life for me and my family* …” (P#13, M, 18) and assist with reaching personal goals (“*My big dream is to be a registered engineer*” P#19, M, 22).

Students’ belief that success is possible, regardless of obstacles, translated aspiration into action. This active sense of hope and self-belief motivated students to seek opportunities for growth and development, leading to tangible achievements, such as obtaining awards or playing leadership roles (“*I learned to never give up. I did not expect a sponsor to help me, but it happened. I was so surprised, but also very thankful. It would not have happened if I just gave up*” P#18, M, 20). From the qualitative analyses, it emerged that students’ successes and achievements validated their continued efforts and reinforced the relevance of hope as a catalyst in pursuing and realising goals “*Lots of things go wrong, but there are small wins along the way*” (P#10, F, 20). When considered through the lens of hope theory and goal-setting theory, this qualitative analysis highlights the relevance of WillPower (agency), WayPower (pathways) and WhyPower (motivation) components as catalysts that can propel students forward, motivating them to pursue their academic goals despite challenges (Bieleke et al., 2020; Colla et al., 2022; Latham and Locke, 2007).

However, participants also revealed that their future ambitions were not restricted to academic achievements alone. Instead, they agreed that their future ambitions encompassed a broader spectrum of meaningful life pursuits and a strong desire to effect societal change. One participant contemplated:

*Getting my qualification will open doors for me. But I want more than that. I would say it is about honouring my legacy. My parents sacrificed a lot, and I have the responsibility to be everything they sacrificed for. There is a quote … it says, ‘I am my grandparents’ greatest hope.’ What it means for me is that they sacrificed and fought many battles for me to be here today at university. It cannot be just about a qualification … is about more than that* [referring to an academic qualification] (P#12, F, 23).

This quote reflects a profound understanding among students of the potential impact their careers and academic studies can have beyond personal success, extending into societal benefit and its roots in sacrifices made by previous generations. However, some participants lamented the generational responsibilities they would need to carry as university graduates. In this regard, participant 16, a 19-year-old male, commented:

As a graduate, you are expected to contribute to your family. You do not simply get a high-paying job or live in luxury. You are indebted to your extended family. For example, you will be asked to pay for your cousin’s child when he goes to university. This thing called black tax holds you hostage.

Other participants agreed (“*Black tax is real… you will be responsible for many family members, and they will hassle you for it*” P#2, F, 19). However, other participants provided an opposing view by indicating, “*Caring and supporting my family is a blessing, and I am glad to be the one who can do that for them… they gave up for me*, *and I can help them after I graduate… it is expected in our culture*” (P#9, F, 19).

The concept of black tax refers to the financial support that many black professionals in South Africa, such as graduates, are expected to provide to their families and extended families, such as parents, grandparents and siblings (Leibbrandt et al., 2012; Mangoma and Wilson-Prangley, 2019). Black tax is often considered a social obligation rooted in communal responsibilities, and the practice has historical roots in colonial and apartheid eras where disenfranchised communities required such support to address socioeconomic inequalities (Leibbrandt et al., 2012). The literature points to complexities and varying perspectives on the topic and practice of black tax. Whereas some regard it as a form of ubuntu in collectivist cultures, others describe it as a continuation of generational poverty based on a legacy of institutional racism (Broodryk, 2006; Mhlongo, 2019). However, it remains true that cultural practices, such as black tax and adherence to ubuntu principles, affect a large segment of South African first-generation students who may, in some instances, be placing their hopes for a better future on obtaining a university qualification (Mhlongo, 2019). Hence, student affairs staff and others, specifically professionals working in student counselling units, should acknowledge the jarring juxtaposition of regarding a university qualification as the avenue to an empowered life versus the added generational burden affecting first-year students who are already facing significant stressors as they transition from school to university.

In light of the above, universities are more than just educational institutions and lecture halls that offer knowledge and competencies required to obtain academic qualifications; they are incubators for hope (Cherrington, 2017, 2018; Maniram, 2022). In this regard, students commented that support programmes and an enabling campus environment are essential for navigating the complexities of current academic challenges and the uncertainties associated with future roles (“*Offering counselling to students is important… way to think through problems and find solutions*” P#14, M, 18). However, universities also have roles in supporting students in reflecting on, addressing, and developing creative yet socially relevant pathways to deal with cultural realities, such as the black tax, which affect their academic journeys. Thus, supporting students in realising hope requires more than WillPower and WayPower; it requires an ecologically nested understanding of the culturally relevant challenges and opportunities that students and others experience (Cherrington, 2017; Colla et al., 2022; Mhlongo, 2019; Scioli et al., 2011).

In conclusion, this theme reveals an expectation among students that universities should play an active role in preparing them for the future, not just through academic instruction but also by fostering an environment that nurtures hope, inclusivity, and a commitment to culturally relevant societal advancement. In this regard, one participant reflected, “*Hope is seeing the future as bright and inviting while knowing the hardships we have endured and the challenges that await us. I am a product of my heritage living in a world with many people and cultures*” (P#11, F, 19). Such an expectation calls for universities to reassess and expand their roles, ensuring they equip students with the tools, knowledge, and support systems necessary to achieve their aspirations and contribute meaningfully to society. As a psychological construct, hope should be explored from various perspectives and drawn on to support students.

## 4 Discussion

This qualitative study revealed the dynamism of hope and challenge among 22 first-year South African university students transitioning from school to university. The qualitative themes highlighted students’ multifaceted perspectives of hope and its affirming role in addressing challenges and future aspirations within a collectivist culture.

First, the students’ perspectives revealed a dynamic duality between the excitement and pride of new beginnings and the anxieties of unfamiliarity and newness of entering higher education. This affective and social duality was particularly prevalent among the 15 first-generation students, who reported facing challenges without the benefit of familial experience and often with added financial burdens. Despite the difficulties reported, students also demonstrated resilience, reframing initial overwhelm as a catalyst for growth.

Second, hope was described as a multifaceted concept and a guiding light, illuminating students’ paths and fostering resilience to persevere despite challenges. Furthermore, hope was described as a driving force that fuelled students’ future ambitions and aided in translating aspirations into tangible achievements. Students regarded a hopeful disposition as crucial for navigating university and other difficulties. The relevance of religion was also highlighted and emerged as a pertinent source of hope. This theme emphasises the dynamic nature of hope and suggests it is a complex lived experience, entailing various dimensions.

Third, hope was considered as extending beyond future academic aspirations and expressed a desire for personal fulfilment and societal impact. Even though obtaining an academic qualification was regarded as a pathway to empowerment, participants also grappled with the weight of generational responsibilities, particularly those associated with black tax. This cultural reality adds a layer of complexity to their pursuit of a brighter future, highlighting the need for universities to acknowledge cultural realities when drawing on hope theory as a framework to support students.

The qualitative themes indicated that university students in South Africa consider hope as a dual force, balancing personal ambition with communal obligations. Snyder’s (2002) dual factors of agency and pathways align with students’ aspirations for academic success. However, students also experience hope within a collective frame rooted in the values of Ubuntu, the responsibilities of *inter alia* black tax and familial support structures (Mangoma and Wilson-Prangley, 2019). Whereas participants articulated hope as a means to manage the initial challenges of university life—moving from feelings of alienation and financial pressure toward empowerment and resilience—their experiences also echo Cherrington’s Afrocentric hope framework, where hope functions as a guiding light amidst adversity. Hence, hope is framed as a pathway to uplift the self and the collective.

This thematic analysis contributes valuable insights into the qualitative experiences of hope among first-year university students in South Africa. The findings have suggested the need for support services that foster hope in culturally sensitive ways that speak to students’ diverse backgrounds. Furthermore, the qualitative analysis revealed that hope is not just a passive desire but an active force shaping students’ attitudes, behaviours, and strategies for coping with university life’s challenges. Moreover, hope resides in the individual and the collective, encapsulating a resilient stance towards life. This dynamic interplay of hope and resilience underpins the unique journey of first-year generation university students, preparing them for future successes and societal contributions.

### 4.1 Limitations

While this qualitative study offers insights into the hopes and challenges experienced by first-year university students, the findings are subject to at least three limitations. First, the study was conducted at a single research site. This single-site limitation implies that the data reflected the local culture, including the university culture. Hence, the findings and qualitative principles may not be transferable to other contexts. It should also be noted that different universities attract students from diverse cultural, socioeconomic, and geographical areas. Future research should collect data from multi-site and diverse settings to explore cross-cultural differences and capture the multifaceted nature of the student experience related to hope across South Africa.

Second, the study was conducted at a specific point in time. This temporal limitation means that the research could not account for the dynamic and evolving aspects of students’ experiences. Various strategies can be adopted to address this limitation in future studies. Amongst others, researchers could adopt a longitudinal approach, thereby collecting data at various points during the academic year or across multiple years during students’ academic journeys. Repeated cross-sectional studies at different time points should be considered to address practical concerns associated with longitudinal research. Alternatively, prolonged engagement with a sample of students could assist in addressing the temporal limitations.

Third, further exploration through theoretical sampling could have provided additional insights and a more nuanced understanding of hope. Theoretical sampling can also provide greater flexibility for following emerging insights. For example, intentionally seeking out students who have experienced significant challenges or exhibited resilience in specific circumstances could have provided a richer understanding of students’ hopes and challenges as they navigate the transition from school to university.

### 4.2 Recommendations

This study underscores the multidimensional nature of hope among South African university students navigating the challenging transition from school to university. Three recommendations and implications are discussed accordingly.

First, investigating hope from non-Western perspectives is an idea whose time has come. The students’ experiences reported in this study underscore how sociocultural factors shape conceptualisations and expressions of hope. Hence, future research should emphasise exploring hope within diverse cultural contexts. Cross-cultural research can offer a more nuanced understanding of hope by providing insight into universal principles through acknowledging diverse cultural experiences and expressions.

Second, the findings have implications for student affairs services. Through recognising the dynamic nature of hope, a shift can be established from focusing mainly on academic support to fostering holistic student success. This includes, *inter alia*, acknowledging the cultural factors shaping students’ experiences and providing culturally relevant and sensitive services. Universities should also prioritise initiatives that cultivate hope, such as peer support networks, early support services and awareness campaigns that incorporate the concept of hope to assist students in building mental buffers that could enhance coping with the inevitable challenges within a university context. Universities can introduce financial aid and support services by recognising the cultural realities and potential financial burdens students may encounter, such as black tax. The findings indicated that addressing financial stressors is crucial to fostering hope and promoting equitable access to higher education.

Third, this study highlights the importance of hope as a theoretical construct for understanding students’ real-life experiences. The findings indicate that hope is not merely a passive desire but a multidimensional force shaping student attitudes and behaviours. The qualitative data revealed hope as a guiding light illuminating paths through challenge, a driving force fuelling ambition, a source of belonging through social connection, and a source of strength enabling students to confront obstacles. This dynamic interplay of hope and resilience underpins the challenging first-year experience.

This study illustrates the potential impact of hope on the first-year experience among South African university students. As champions of hope, we can empower first-year university students to navigate the labyrinth of challenges by embracing multicultural perspectives on hope, implementing hope-informed support services, and recognising its significance as a theoretical construct with real-life implications.

## 5 Conclusion

This research underscores the role of hope as a multifaceted construct that transcends individual aspirations to encompass broader communal objectives, especially within African contexts. While Western hope theories emphasise agency and pathway mechanisms for personal success, African conceptualisations integrate hope with values of interdependence, responsibility, and community.

First-year university students in South Africa navigate academic pressures alongside responsibilities to family and community. Recognising this dual nature, the study highlights the importance of culturally nuanced support structures within universities, empowering students to harness hope as a resilient force that serves individual and societal development.

This qualitative study indicated that hope is a lived reality and a resilient stance that students can adopt in facing life’s inevitable challenges. Hope was conceptualised as a symbolic bridge nested in socio-cultural realities, leading from the present to a future filled with unimagined possibilities. For South African students, hope epistomes their unique long walk to freedom as they transition from school to university and beyond, dreaming of and realising a better tomorrow.

## Data Availability

The raw data supporting the conclusions of this article will be made available by the authors, without undue reservation.
